# Supplementary material for: Stop and think: Additional time supports monitoring processes in young children
Source: PLoS One. 2022 Sep 15;17(9):e0274460. doi: 10.1371/journal.pone.0274460 (PMC9477363; doi:10.1371/journal.pone.0274460)
Supplement: Attachment — Submitted filename: Response_to_Reviewers.docx [file pone.0274460.s003.docx]

# **Rebuttal Letter**

**Thank you for the opportunity to revise my paper. I appreciated the comments and the interesting inputs to improve the quality of the present study.**

My Comments appear in blue

Journal Requirements:

1. When submitting your revision, we need you to address these additional requirements.

Please ensure that your manuscript meets PLOS ONE's style requirements, including those for file naming. The PLOS ONE style templates can be found at

https://journals.plos.org/plosone/s/file?id=wjVg/PLOSOne_formatting_sample_main_body.pdf and

<https://journals.plos.org/plosone/s/file?id=ba62/PLOSOne_formatting_sample_title_authors_affiliations.pdf>

I ensured that my revision does meet the PLOS ONE's style requirements.

2. You indicated that you had ethical approval for your study. In your Methods section, please ensure you have also stated whether you obtained consent from parents or guardians of the minors included in the study or whether the research ethics committee or IRB specifically waived the need for their consent.

I have added the following paragraph to the method section. (Lines: 171 – 176)

The Ethics Committee of the Faculty of Human Sciences at the University of Bern approved ethical consent for the study (Approval No: 2002-100005). The parents or legal guardians of all participating children signed an informed consent. Further, all children were asked verbally to participate prior to the testing. They were further explained that they could terminate the task at any time. No child ever did. Data is entirely anonymous.

3. We note that you have indicated that data from this study are available upon request. PLOS only allows data to be available upon request if there are legal or ethical restrictions on sharing data publicly. For more information on unacceptable data access restrictions, please see [http://journals.plos.org/plosone/s/data-availability#loc-unacceptable-data-access-restrictions](http://journals.plos.org/plosone/s/data-availability#loc-unacceptable-data-access-restrictions).

The data of the present study is available. Data Availability Statement: All relevant data for the manuscript are submitted within the Supporting information files. (2 Excel files: Data Monitoring: S1_File.excel; Data Inhibition: S2_File.excel)

In your revised cover letter, please address the following prompts:

a) If there are ethical or legal restrictions on sharing a de-identified data set, please explain them in detail (e.g., data contain potentially sensitive information, data are owned by a third-party organization, etc.) and who has imposed them (e.g., an ethics committee). Please also provide contact information for a data access committee, ethics committee, or other institutional body to which data requests may be sent.

The data of the present study is available. Data Availability Statement: All relevant data for the manuscript are submitted within the Supporting information files. (2 Excel files: Data Monitoring: S1_File.excel; Data Inhibition: S2_File.excel)

b) If there are no restrictions, please upload the minimal anonymized data set necessary to replicate your study findings as either Supporting Information files or to a stable, public repository and provide us with the relevant URLs, DOIs, or accession numbers. For a list of acceptable repositories, please see http://journals.plos.org/plosone/s/data-availability#loc-recommended-repositories.

We will update your Data Availability statement on your behalf to reflect the information you provide.

Minimal anonymized data sets necessary to replicate the present study findings are added.

4. Please include your full ethics statement in the ‘Methods’ section of your manuscript file. In your statement, please include the full name of the IRB or ethics committee who approved or waived your study, as well as whether or not you obtained informed written or verbal consent. If consent was waived for your study, please include this information in your statement as well.

I added the required statements in the methods section.

5. Please review your reference list to ensure that it is complete and correct. If you have cited papers that have been retracted, please include the rationale for doing so in the manuscript text, or remove these references and replace them with relevant current references. Any changes to the reference list should be mentioned in the rebuttal letter that accompanies your revised manuscript. If you need to cite a retracted article, indicate the article’s retracted status in the References list and also include a citation and full reference for the retraction notice.

I have checked the reference list. Compared to the first submission, I have added five additional articles to the manuscript and reference list. I marked them in the manuscript (marked-up copy) correspondingly. However, none of these articles have been retracted.

**Comments to the Author**

1. Is the manuscript technically sound, and do the data support the conclusions?

The manuscript must describe a technically sound piece of scientific research with data that supports the conclusions. Experiments must have been conducted rigorously, with appropriate controls, replication, and sample sizes. The conclusions must be drawn appropriately based on the data presented.

Reviewer #1: Partly

Reviewer #2: Partly

Reviewer #3: Yes

2. Has the statistical analysis been performed appropriately and rigorously?

Reviewer #1: I Don't Know

Reviewer #2: Yes

Reviewer #3: Yes

3. Have the authors made all data underlying the findings in their manuscript fully available?

The [PLOS Data policy](http://www.plosone.org/static/policies.action#sharing) requires authors to make all data underlying the findings described in their manuscript fully available without restriction, with rare exception (please refer to the Data Availability Statement in the manuscript PDF file). The data should be provided as part of the manuscript or its supporting information, or deposited to a public repository. For example, in addition to summary statistics, the data points behind means, medians and variance measures should be available. If there are restrictions on publicly sharing data—e.g. participant privacy or use of data from a third party—those must be specified.

Reviewer #1: Yes

Reviewer #2: No

Reviewer #3: No

4. Is the manuscript presented in an intelligible fashion and written in standard English?

PLOS ONE does not copyedit accepted manuscripts, so the language in submitted articles must be clear, correct, and unambiguous. Any typographical or grammatical errors should be corrected at revision, so please note any specific errors here.

Reviewer #1: Yes

Reviewer #2: Yes

Reviewer #3: Yes

5. Review Comments to the Author

Responses to reviewers are documented below.

# **Response to Reviewer #1**

My comments appear in blue

Reviewer #1: In this manuscript Wacker and Roebers investigated the discrepancy between children’s monitoring abilities in day-to-day life, which appear to be pretty good, and their monitoring abilities in research settings, which appear to be poor. They tested if giving children extra time before self-reporting their monitoring (i.e., confidence judgements) increased monitoring accuracy in a research setting and if these self-reports were related to inhibitory control. The manuscript was succinct and straightforward and made it clear that this study was motivated by previous research. Furthermore, the ideas/predictions were easy to follow. I only have a few clarifications or methodological/statistical related comments.

Thank you for your helpful comments and for giving me the opportunity to revise the manuscript. I have been able to understand your inputs and adapted them accordingly. You’ll find my answers to your comments in blue.

1. One major issue is that the data appear to be all between subjects (based on the methods section) and yet some of the information in the results section makes it seem like some data was being treated as within subjects. I’m assuming this is a typo and that the correct test was run (degrees of freedom numbers in results section suggest a between subjects ANOVA), but it does need to be corrected in the text.
• Methods section text: “Children were randomly assigned to either the control group (CG); they solved the task as described above or the experimental group (EG).”
• Fig 1 and Fig 2 text: “Note. Discrimination Score, between factors Age (Kindergartners vs. Second Graders), within factors Condition (Control Group (CG) vs. Experimental Group (EG))”
Related to this issue, please indicate what type of ANOVA you are running (between-subjects, within-subjects, or mixed) when you describe your ANOVA based analyses (currently the manuscript only says that it was a 2x2 design).

Thank you for your comments. I have added some more details about the design and the type of the measures (ANOVA) and adjusted the descriptions of the figures (statistical analysis and result section).

2. The last sentence in the abstract, “The pattern of results revealed an interactive role between monitoring and inhibition only for children in kindergarten and not for second graders”, is not fully supported by the results. There was a correlation done between monitoring and inhibitory control for each age group and so you could say that there was a correlation between monitoring and inhibition for kindergarteners. However, unless you directly test an interaction between monitoring and inhibitory control and include both age groups in this test, you cannot say that there was an interaction between monitoring and inhibitory control only for one age group.
• Nieuwenhuis, S., Forstmann, B. & Wagenmakers, EJ. Erroneous analyses of interactions in neuroscience: a problem of significance. Nat Neurosci 14, 1105–1107 (2011). <https://doi.org/10.1038/nn.2886>
Moreover, the correlations between monitoring and inhibitory control were very close in their r values (0.225 for kindergarteners and 0.200 for second graders) despite them only being significant for kindergarteners (p<.05 vs p=.104). An article by Tamar and Jean-Jacques (2019) suggests that a lack of significance can sometimes be due to the sample being underpowered and researchers should be cautious in over interpreting insignificant results. In their article, they do say that a small effect size means an effect is unlikely to be theoretically meaningful. However, I bring up their article because the r values are so close in size between these two groups and I noticed that the kindergarteners are a larger sample than the second graders (n=88 and n=67 respectively). It makes me wonder if the effect for the second graders was non-significant only because the group was smaller and therefore the statistical power was reduced compared to the kindergarten group.
• Makin, T. R., & de Xivry, J. J. O. (2019). Science Forum: Ten common statistical mistakes to watch out for when writing or reviewing a manuscript. Elife, 8, e48175. DOI: 10.7554/eLife.48175

Because our data collection was carried out during Covid-19, due to children in quarantine and the corresponding dropout, we could not capture the H&F task from everyone. Therefore, these considerations addressing unequal sample size and the interpretation of insignificant results are interesting, and I have included these reflections in the discussion (lines: 392 - 396). Thank you for this input. I have also adjusted the abstract section (Lines: 34-36).

3. I also have a minor suggestion concerning how the ACC is discussed in the introduction.
“To engage in monitoring, the responsible neural networks need time to loop signals from the anterior cingulate cortex (ACC) to frontal structures [25] and to trigger a feeling of uncertainty which may lead the child to overthink his confidence judgments.”
When talking about neural networks and signals, it would be better to keep the description simple and not make conjecture about what those neural signals mean in terms of what the subject is thinking or feeling.

Good point, thank you. Mainly because we could not measure cognitive signals during our task, we must be careful in interpreting such potential neurological signals and their relation to other cognitive concepts.

Nevertheless, ample evidence shows that frontal structures and the ACC may be seen as a neurological correlate of monitoring. We also never tried to describe this relation and neurological transmission as a fixed process for the feeling of uncertainty; instead, we used the words like "may" to underline this possible neurological transmission under consideration of the neurological studies. However, I agree that the application and the description should be discussed more carefully, and this association may be expressed simpler. I have adjusted the corresponding part (Lines: 91-96).

4. The text has minor grammatical errors throughout. Nothing major, but I recommend proofreading again before re-submission.

Thank you for your advice. The re-submission has now been proofread again.

# **Response to Reviewer #2**

My comments appear in blue

Reviewer #2: Overall, I think the study provides some interesting data concerning the effect of delaying CR on monitoring accuracy. That being said, this is a pretty well-worn effect in the non-developmental metacognition literature and that should be better acknowledged in the paper.

Thank you for your comments and the possibility to review this paper.

Introduction


There’s a pretty large literature on the accuracy of delayed metacognitive ratings compared to immediate – see the delayed judgment of learning effect (Nelson & Dunlosky, 1991) - which hasn’t been addressed in the introduction and (at least from a non-developmental perspective) might limit the novelty of the findings

Even though Nelsons and Dunlosky's study differs from our design, their results support indirect evidence that time may play a crucial role in monitoring accuracy. I have included their results in the introduction section. Thank you for this comment. (Lines: 103 - 106).

The other literature that seems relevant but isn’t included is the idea of confidence ratings being informed by post-decision accumulation of evidence (e.g., Navajas et al., 2016)– so rather than improved accuracy as a result of inhibiting the CR by a short time, the accuracy might improve because the evidence in favour or against the decision has had longer to accumulate

The study from Navajas et al. (2019) revealed exciting insights between giving an answer and rating a confidence judgment on this answer. I would argue that it may also be an interactive role - while inhibition is needed to take more time to allow further processing and accumulation, monitoring processes may supervise these procedures. Because monitoring processes have to be active to detect and make use of the added information during this interval because without - there may be a change, this potential of further processing may not even be detected. I have integrated their findings in the introduction and the discussion section. (Lines: 114 – 121, 352 – 359)

Results

Could the authors include the performance results as a function of experimental group, it would be good to rule out whether the ‘stop-and-think’ procedure improved performance (which might inflate the monitoring accuracy if kids tend to be overconfident). It seems to indicate in the discussion that stop-and-think can impact performance so better monitoring accuracy due to an artefact of better performance needs to be ruled out

Thank you for this comment. In the result section, I added the corresponding analysis addressing performance accuracy as a post hoc test. (Lines: 252 – 259).

Unless I misunderstood both groups completed the inhibition task, so why not include both groups and look at the interaction between group X inhibition predicting accuracy. It would, for example, tell you whether the intervention was more effective for children with low inhibition

Both the EG and the CG have completed the H&F task. In our comparison, however, we intentionally compared only the inhibition of the CG with the MC task because we were interested in exploring whether individual differences in inhibition are related to monitoring accuracy independently from our manipulation. However, I agree that the inclusion of the EG may reveal interesting additional information. This allows us to address whether inserting the delay between recognition and monitoring has a differential effect on participants depending on their inhibitory control skills. I included the analysis in the results section (Lines: 259 – 269, 319 – 325).

Discussion

A few of the points above should also be incorporated into the discussion, especially when trying to outline possible mechanisms why more time is helpful for metacognition

Information added.

In terms of the limitation you raise, as a future study, it might be interesting to think about the stop-and-think vs some other delay – so rather than explicitly cueing more metacognitive thought simply allowing more time to reflect in an unguided way

Information added. (Lines: 434 – 436)

Minor

Line 75 – I would remove the tail end of the sentence “according to the often used instruction ‘stop and think’”, it’s not necessarily a familiar manipulation to people not in this sub-field

I have adjusted the corresponding part.

Report the exact p-value on line 233, line 248, line 264 etc (i.e. don’t write p < .05)

The exact p values have been added.

For the figures, need a description of the what the boxes indicate. I would add a dotted horizontal line at the zero mark

I have added the information.

The figure captions indicate that the error bars are standard errors of the mean. Is this correct? – they seem extremely wide from my cursory glance if the SDs reported in paper are correct, the SE should be smaller than the SD, if I remember correctly).

Thank you for bringing this to my attention. I have adjusted the description of the legend. The visualization displays a boxplot with corresponding whiskers. The whiskers indicate the min/max value within the definition of a whisker's length (1.5* interquartile range) for the dependent variables. Data points outside of this interquartile range are defined as outliers.

# **Response to Reviewer #3**

My comments appear in blue

Reviewer #3: This paper investigates the role of inhibition on metacognitive monitoring, taking two approaches. The authors manipulated whether participants received a delay before making a confidence judgment, finding that children showed greater monitoring accuracy after the “Stop and Think” manipulation. In addition, the authors found that individual differences in inhibitory control were related to metacognitive monitoring accuracy for younger children but not older children. I believe this study addresses a theoretically important issue - understanding the relation between metacognitive monitoring and executive function, as well as the development of this relation.

Thank you for your positive feedback addressing the importance of our study.

Overall, the paper is clear and well-written. Below I outline some questions and suggestions to enhance the clarity of the paper:
The authors use binary pronouns throughout the paper, with multiple references to “him/his” without any other gender option. Use of binary pronouns can exclude those who are non-binary or who hold other gender identities. I would encourage the authors to use gender neutral language (they/them/theirs) throughout the paper. The APA supports the use of the singular they in academic writing.

Thank you for bringing this to my attention. I have changed the pronouns to a gender-neutral language.

In the method section, the authors mention that the Hearts and Flowers task was used to measure inhibition and cognitive flexibility. However, no description of this task is offered. I would encourage the authors to include a detailed description of the task protocol.

Because our primary interest lies in the measurement of monitoring, and we created a new task capturing monitoring skills, we wanted to ensure that the procedure of this novel task was described in detail. The Hearts and Flowers Task was already applied several times in other studies. Because of the frequent use in the research field of executive functions, we diminished the Hearts and Flowers task's description in our method section. For replication, we indicated the primary literature to provide the connection to the studies using the Hearts and Flowers task. However, I agree that it may confuse the reader why one task is deeply explained and the other not. I, therefore, inserted a short description of the Hearts and Flowers task, especially with additional information about technical and methodological details. (Lines: 229 – 235).

In the results section (line 206-207), the authors mention that participants were excluded if overall accuracy was lower than .50. This is presumably because that would show a response bias in the task. I would encourage the authors to spell that out for the reader.

Information added.

On a theoretical level, it is possible that imposing a greater delay encourages further processing in the base-level task, rather than greater deployment of metacognitive processes. Further processing in the base-level task may make metacognition easier, perhaps reducing the effort required to monitor the base-level task. In other words, the delay may contribute to a stronger signal from the base-level task, which would require less metacognitive effort to detect. I would encourage the authors to consider this possibility in their revisions.

Thank you for this interesting comment. I have integrated your input in the introduction and discussion section. (Lines: 114-121, 352-359).

There were a couple of sentences where the writing/word choice was unclear:
On lines 90-92, the sentence ends with, “... which may lead the child to overthink his confidence judgments.” The use of “overthink” often has negative connotations, but I think the authors are trying to describe a situation in which typical monitoring is occurring.

Good point! We do not associate "overthinking" with a negative context. We wanted to describe how this "stop and think" condition may trigger a feeling of uncertainty where children may hesitate and "overthink" or "reassess" their given confidence judgment. The term "overthink" describes monitoring processes where children evaluate their answers and give them a second thought.

On lines 341-342, the authors write, “A meta-analysis showed that comparing different tasks for measuring inhibitory control is specific to a given age range.” This is unclear and could be easily clarified by including more detail about this finding.

I added some information for clarification.
