# Supplementary material for: Stop and think: Additional time supports monitoring processes in young children
Source: PLoS One. 2022 Sep 15;17(9):e0274460. doi: 10.1371/journal.pone.0274460 (PMC9477363; doi:10.1371/journal.pone.0274460)
Supplement: Attachment — Submitted filename: Response to Reviewers.docx [file pone.0274460.s004.docx]

**Rebuttal Letter**

Thank you for the input addressing my paper in order to clarify and to improve the quality of the present study.

My Comments appear in blue.

**Response to Reviewer #1 and #2**

Reviewer #1: All of my previous comments/suggestions have been addressed. Thank you to the authors for their edits and replies to my comments/suggestions. I believe the manuscript should be accepted for publication.

Reviewer #2: The authors have addressed all of the comments from my earlier review. The paper should make an interesting contribution to the literature.

Thank you for your positive feedback!

**Response to Reviewer #3**

Reviewer #3: I think that the authors responded to all of the reviewers' comments from the original submission. The research is sound, but the manuscript still has some issues in terms of clarity and organization. Below, I outline some suggestions for improvement:

Thank you for the acknowledgment of my improvements. I thank you for your further recommendations to improve the quality of my paper.

In lines 52-56, the authors introduce the process of monitoring, and equate it with confidence and performance estimation. The authors should be clear that these are just 2 examples, and that there are other forms of monitoring (e.g., judgments of learning, feeling of knowing, difficulty estimations, etc.).

I have included a paragraph to draw the reader's attention to the fact that there are also some other measures of monitoring processes (lines: 53-57).

In the paragraph that spans lines 91-121, the argument the authors are trying to make is unclear. They discuss neurological underpinnings of monitoring, indirect evidence of this, the role of increased time on performance and accuracy, and metacognitive processing post-decision. It seems that the paragraph should be organized to make a more direct argument, or it may be split into several paragraphs to make several different arguments

Thank you for your comment. I organized and split the part into several paragraphs to enhance clearness (lines: 95 – 128).

In lines 99-101, the authors suggest that part of the monitoring process involves asking "Am I really sure about my answer?" Previous work has investigated both explicit and implicit forms of metacognitive monitoring, so the authors should clarify their viewpoint on the role of conscious processing in metacognitive monitoring in young children

I have added some more information (line: 106 - 109).

In addition, in the discussion of previous research on what happens during the "time to loop signals from the ACC to frontal structures", I suggest the authors clarify what is happening functionally during this process, and what the average processing time is, as these seem relevant to the manipulation in the current study

The procedure and measure section explains how long we set our delay (lines: 217 - 223). Because this is a novel approach, and to our knowledge, no studies so fare exist addressing this issue, we do not have any additional references. Our delay builds on the idea of neurological diffusion (about 200-250 ms) as well on studies implementing a delay in other cognitive tasks. We argue that firstly additional time for diffusion of neurological signals and secondly time for monitoring processes result in a reasonable delay. Further research is needed to identify the best processing time range. Nevertheless, our study contributes the first insight regarding a delay for monitoring and may be a guiding reference point for further studies.

The authors write, "The engagement with uncertainty may trigger cognitive processing." This is unclear, did the authors potentially mean "may trigger metacognitive processing"?

I have added some information for clarification (lines: 108 – 109).

Later in the paper, the authors write, "Inhibiting the prepotent response allowing neurological signals to spread out…" What is meant by "spread out"? I noticed that there were no citations next to this claim - what is the previous research that suggests this?

By using the term "spread out" we are addressing the diffusion of neurological signals. These neurological signals emerge at a point x and subsequently diffuse or, as we call it, "spread out".

I have changed the wording and I also added citations (lines: 126 – 128).

In line 124, the authors write, "stop and think! About the answer profoundly". As they mention later in the manuscript, it is not guaranteed that participants thought about the answer profoundly, so I would encourage the authors to reconsider the wording here.

Thank you for your comment. I have adapted the corresponding part (line: 131).

In line 127, the authors should clarify whether they believe the manipulation will positively affect children's monitoring skills (implying the acquisition of new strategies and potentially stable improvement) or their monitoring performance (implying temporary, in the moment improvements).

I added that we are only expecting temporary and, therefore, in the moment improvements (line: 138). However, further research is needed to evaluate if more practice with a "Stop and Think" condition would also lead to an acquisition of a “Stop and Think” as a metacognitive strategy and be also transferable to other domains.

In line 134, the authors write, "We did not expect any effect on recognition". At this point in the manuscript the recognition task has not been described. I would suggest a slightly more detailed description of the base-level task in this paragraph.

I have added some information regarding the task (lines: 131 –136).

In lines 177-178, the authors write "Additionally, during one test session, N = 20 children had to quarantine due to COVID-19." I would encourage the authors to explain why these children needed to be excluded. Was it due to incomplete data/ Which portions of the experiment did they complete or fail to complete?

These children did not solve the paired associate learning task. Due to the restrictions in the school setting we were not allowed to retest them.

I have added some information (lines: 189 – 191).

One interesting finding was that there was a correlation between monitoring and inhibition in kindergarteners only in the control condition. I suggest that the authors provide a potential explanation for this finding in the Discussion section.

These results are discussed in the discussion section (lines: 398 - 430).

In lines 336-338, the authors write, "In absolute terms, children were able to discriminate substantially between correct and incorrect responses". Was there an analysis described in the Results section that directly tested this? Were the discrimination scores significantly different than 0?

Yes, this marked sentence is one of our main findings regarding monitoring. The corresponding analysis is in the result section (ANOVA with dependent variable: discrimination score). Discrimination of confidence judgments between correctly and incorrectly solved items) and the bias index (under- and overestimation).

In the Implications paragraph, I think the authors could include a more detailed and compelling description of the real-world implications of "stopping to think." Where might this come into play for both children and adults? In addition, the sentences "Also, from a neurological perspective, we would expect that neural signals generated from the ACC transferred to frontal regions need time for transmission. In other words, the metacognitive neurological signal then has time to spread out and influence monitoring processes." do not seem to be an implication of the current study, but speculation about the neurological processes involved. While appropriate for the discussion section, I do not think this belongs in the implications section.

I have added some information (lines: 438 - 441).
